# Enhancement of object detection with transcranial direct current stimulation is associated with increased attention

**DOI:** 10.1186/1471-2202-13-108

**Published:** 2012-09-10

**Authors:** Brian A Coffman, Michael C Trumbo, Vincent P Clark

**Affiliations:** 1 The Department of Psychology, University of New Mexico, MSC03-2220, Albuquerque, NM 87131-1161, USA; 2 The Department of Neurosciences, University of New Mexico, MSC08-4740, Albuquerque, NM 87131-5223, USA; 3 Clinical Neuroscience Center, Dept. Psychology, University of New Mexico, MSC03-2220, Albuquerque, NM 87131-1161, USA; 4 The Mind Research Network and Lovelace Respiratory Research Institute, 1101 Yale NE, Albuquerque, NM 87106, USA

**Keywords:** Learning, tDCS, Frontal Cortex, Object detection, Visual search, Brain stimulation, Attention, Alerting, Attention networks test

## Abstract

**Background:**

We previously found that Transcranial Direct Current Stimulation (tDCS) improves learning and performance in a task where subjects learn to detect potential threats indicated by small target objects hidden in a complex virtual environment. In the present study, we examined the hypothesis that these effects on learning and performance are related to changes in attention. The effects of tDCS were tested for three forms of attention (alerting, orienting, and executive attention) using the Attention Network Task (ANT), which were compared with performance on the object-learning task.

**Results:**

Participants received either 0.1 mA (N = 10) or 2.0 mA (N = 9) tDCS during training and were tested for performance in object-identification before training (baseline-test) and again immediately after training (immediate test). Participants next performed the Attention Networks Task (ANT), and were later tested for object-identification performance a final time (delayed test). Alerting, but not orienting or executive attention, was significantly higher for participants receiving 2.0 mA compared with 0.1 mA tDCS (*p* < 0.02). Furthermore, alerting scores were significantly correlated with the proportion of hits (*p* < 0.01) for participants receiving 2.0 mA.

**Conclusions:**

These results indicate that tDCS enhancement of performance in this task may be related in part to the enhancement of alerting attention, which may benefit the initial identification, learning and/or subsequent recognition of target objects indicating potential threats.

## Background

TDCS has become increasingly popular for applications in clinical and neurocognitive research, with a broad range of effects and effect sizes. TDCS has shown promise for the treatment of depression [1] and stroke [2], and has been documented to produce cognitive enhancement in healthy subjects in a large number of recent studies: TDCS has been shown to facilitate working memory [3], motor learning [4-7], simple somatosensory and visual motion perception learning [8], and memory for word lists [9].

We have previously found that tDCS can increase learning in a complex visual search task involving detection of target objects hidden in a complex virtual environment that indicate possible threats [10-12]. In these studies, participants were trained to classify those images as target object present or target object absent. Participants received anodal tDCS at up to 2.0 mA using 11 cm^2^ electrodes during training with the electrode positioned over 10–10 EEG position F10 (over the right sphenoid bone above inferior frontal cortex). Though no differences were present at baseline in these studies, large improvements in performance occurred for participants receiving 2.0 mA tDCS. Similar to results presented by Iyer et al. [13], these effects were dose-dependent, with performance improvement showing a highly linear relationship with current strength.

It is unclear from our previous studies what, specifically, mediates the effects of tDCS on learning and performance of this task. We have found that a combined measure of glutamate and glutamine (Glx), and also N-acetyl aspartate (NAA) are all increased by tDCS under the anodal electrode, but not in the opposite hemisphere [14], which may result in increased neural activity and plasticity, resulting in greater learning. However, another possibility is that tDCS alters functioning in attentional/perceptual networks in such a way as to enhance the perception of targets, leading to greater learning by virtue of greater perceptual acuity. The specific attentional processes that may be involved in this affect are uncertain.

Here we collected three measures of attention using the Attention Networks Test (ANT) [15] in effort to identify the extent to which attention is modified by tDCS over the right inferior frontal cortex. These data were collected along with performance measures on the target learning task reported previously by our lab [11]. The ANT is a combination of the cued reaction time (RT) task [16] and the flanker task [17], and it requires participants to indicate with a speeded button press response whether a centrally presented arrow points to the left or to the right. Efficiency of three attention networks are assessed by measuring how RT is influenced by alerting cues (assessing the alerting network), spatial cues (assessing the orienting network), and flankers (assessing the executive network). These three measures have been validated in previous behavioral and imaging studies and represent functionally and anatomically distinct attention networks in the brain [18,19]. Alerting is defined as achieving and maintaining an alert state and has been linked to right hemisphere fronto-parietal brain networks [20]; orienting measures the efficiency of information selection from sensory input and is mediated mainly by superior parietal cortices and frontal eye fields [21]; and executive control, a measure of conflict resolution among conflicting visual stimuli, is mediated by medial and lateral frontal cortex [22]. We hypothesized that measures of alerting, orienting, and/or executive attention would be enhanced with tDCS, and that increased attention in this/these enhanced domain(s) of attention would be positively correlated with object detection scores in participants receiving active (2.0 mA) but not sham (0.1 mA) tDCS.

## Results

**Figure 1:**
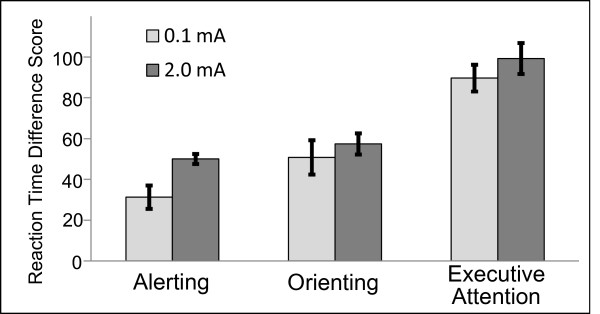
***Effects of tDCS on Alerting, Orienting, and Executive Attention. ***Participants receiving 30 minutes of 2.0 mA anodal tDCS over right inferior frontal cortex (dark bars) had significantly greater alerting scores from the ANT than those receiving 0.1 mA (light bars). Differences between tDCS groups for scores of orienting attention and executive attention were nonsignificant. Error bars represent standard error of the measurement.

**Figure 2:**
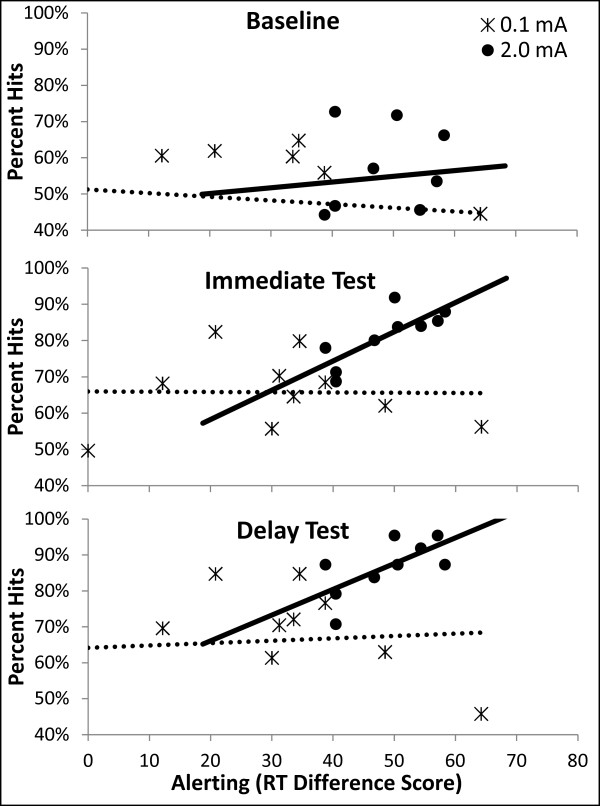
***Correlation between Alerting Scores and Percent Hits at Baseline, Immediate, and Delay Tests. ***Alerting scores were significantly correlated with the percent of hits in the immediate and delayedtests for participant receiving 2.0 mA tDCS (solid lines). There was no correlation for participants receiving 0.1 mA tDCS (dotted lines), nor was there a significant correlation for either group between alerting and percent hits during baseline testing. Four data points below 40% are not visible in the baseline figure (top).

There were no significant differences between tDCS groups in age, years of education, gender, ratio of hours slept prior to experimentation to hours normally slept, hours spent per day playing video/computer games, or number of coffee drinkers (all *p*’s > 0.4). TDCS significantly affected participant’s alerting attention (*F*(1,17) = 7.054; *p* = 0.017), with greater alerting reaction time (RT) difference scores for participants receiving 2.0 mA (49 ± 2 ms; mean ± SEM) than those receiving 0.1 mA (31 ± 6 ms; Figure 1). The variance in alerting RT scores for participants receiving 2.0 mA was not significantly different from participants receiving 0.1 mA, as determined by Levene’s test for equality of variances (*F*(17) = 2.92; *p* = 0.148), suggesting that there was no significant effect of tDCS on variance of response times. There was no statistically significant effect of tDCS on orienting or executive attention (both *p*’s > 0.1). Further analyses indicated that, for participants receiving 2.0 mA tDCS, alerting scores were significantly correlated with the proportion of hits in the immediate test (*r* = 0.790; *p* = 0.011; Figure 2) and the delayed test (*r* = 0.848; *p* = 0.004; Figure 2), but were not correlated with hits at baseline (before training began). None of these measures were correlated with alerting for participants receiving 0.1 mA tDCS. The proportion of false alarms, *d*’, and β were not correlated with alerting in either tDCS group (all *p*’s > 0.1).

There was a significant difference in reported skin sensation during tDCS between tDCS groups for the self-reported measure of heat (*t*(17) = −2.694; *p* = 0.015), but the difference was small and no participant receiving 0.1 mA tDCS reported heat sensation above the minimum possible score (i.e. 1, out of a 10 point scale), leading to a lack of variance within that group (0.1 mA = 1 ± 0; 2.0 mA = 2.2 ± 1.4). Itching and tingling were not significantly different between groups and no measure of skin sensation was correlated with any measure of attention (all *p*’s > 0.1). No measure of mood collected at the end of the experiment was significantly different between tDCS groups and mood measures were not correlated with any measure of attention (all *p*’s > 0.1).

## Discussion

As we have previously reported, 2.0 mA of anodal tDCS over right inferior frontal cortex significantly increased performance with training of a difficult hidden object detection task. In addition, we found that higher tDCS current was associated with increased measures of alerting attention. Furthermore, the proportion of hits was strongly and significantly correlated with attention scores for participants receiving 2.0 mA tDCS. This suggests that tDCS enhancement of object detection performance is in part related to enhancement of alerting measures of attention.

One possibility is that enhancement of alerting attention by tDCS led directly to an increased ability to detect objects in the immediate and delayed tests. A heightened state of alertness during testing would be expected to enhance participants’ ability to identify threatening objects hidden within the complex scenes that were used in this task. In this study we targeted the right inferior frontal cortex, a prominent node of the fronto-parietal attention network thought to support alerting attention as measured by the ANT. Enhancement of alerting attention means that, (1) after 2.0 mA tDCS, participants were quicker to respond to the cue stimulus within the 100 ms interval between cue and target onsets, resulting in a faster response to target stimuli, when compared with 0.1 mA controls, or (2) participants receiving 2.0 mA tDCS were able to achieve a heightened state of alertness overall, with greater priming of target perception and/or response. Enhancement of object detection could be explained to some degree by either or both of these interpretations of these results. If participants are faster to achieve a state of cue-induced alertness, then they might have more time to search for threat stimuli in the test images. This is unlikely to have led to the large increases in learning present in this study, however, as participants were given 2 seconds to view the images, and differences in alerting reaction time scores were on the order of 20 ms, or 1/100 of the stimulus duration in the object detection task. Alternatively, a continuously heightened state of alertness could speed processing of stimuli within the complex virtual environment in this task, leading to the ability to process a greater number of objects in the image and/or more time to consider the response to the image before the end of the response window. Another possible explanation for these results is modulation of perception. Perhaps tDCS enhanced perception for both the object detection task and the ANT, leading to greater performance on both measures. If this were the case, however, one might expect that orienting and executive attention would differ between tDCS groups. Further research is needed to disentangle these possible cognitive effects of tDCS over right inferior frontal cortex.

The frontal-parietal attention network assessed by the alerting measure of the ANT has been proposed by Coull et al. [23] to be associated with vigilance in continuous performance tasks and has been specifically implicated in sustained attention during object selection [24]. TDCS may have prolonged sustained attention in this study, leading both to greater hit rate and alerting reaction time scores on the ANT task. Given that the immediate test and ANT were performed *after* approximately 1.5 hours of experimentation, it is possible that the differences here can be explained in part by differences in the fatigue between the groups receiving 0.1 mA and 2.0 mA tDCS. However, we did not find that the self-reported measure of fatigue assessed by our mood/state questionnaire was related to enhancement of alerting or that it differed between tDCS groups. Future studies might benefit from examining the relationship between tDCS of the right inferior frontal cortex and sustained attention more directly, using other attention tasks aside from the ANT.

Interestingly, no correlation was found between the alerting attention measure of the ANT and false alarm rate or *d’.* Our previous studies [11,12] have shown that tDCS significantly reduces false alarm rate and increases *d’*. It is likely that the results of these previous studies can be explained by tDCS effects on multiple cognitive domains, each of which may account for effects on different performance measures for this task. *d’* is calculated by taking the difference between normalized hit rate and normalized false alarm rate. When considered here, this suggests that the nonsignificant relationship between alerting and *d’* might be explained by a nonsignificant relationship between alerting and false alarm rate specifically. Perhaps tDCS enhancement of learning and memory during training led to a greater understanding of object identities and general rules of object locations, increasing participants’ *d*’ scores and decreasing false alarms, while enhancement of alerting led to greater achievement and maintenance of visual search performance during the object detection task, but did not improve the false alarm rate. Another possibility is that effects of tDCS on false alarm rate result from increased risk aversion. Fecteau et al. [25] show that anodal tDCS near the region targeted in this study decreases risky behavior in the Balloon Analog Risk Task (BART), despite incentive for risky behavior. In this context, more risk-averse participants might be more cautious in responding to ambiguous stimuli. This would lead to a lower proportion of object-present responses in trails where no object was detected by the participant.

The ANT has previously been used to examine the relationship between attention and various psychiatric and neurological disorders, including borderline personality disorder [26], dyslexia [27], schizophrenia [28], attention-deficit hyperactivity disorder [29], and depression [30]. Deficits in alerting as measured by the ANT have been found for elderly individuals relative to a younger population [31], and alerting scores have been found to vary by subtype of ADHD [32]. Perhaps enhancement of alerting through modulation of the fronto-parietal alerting network with tDCS might reduce deficits found in these populations. Research into the application of tDCS to these clinical issues could lead to new treatments and interventions without the necessity of pharmacological therapy.

### Caveats

While this study demonstrates compelling evidence of tDCS effects on basic measures of attention, there were several limitations which should be mentioned. We did not collect baseline alerting scores in this study and it is possible that differences demonstrated between participants receiving 0.1 and 2.0 mA tDCS were due to pre-existing differences in alerting. This is unlikely, however, as there was no correlation between alerting scores and measures of performance on the object detection test performed before training began, and there was no difference in object detection at baseline. Also, the design of this experiment was chosen to maximize the effects on object detection performance by placing the anodal electrode over right inferior frontal cortex, but future studies examining the effects of tDCS on measures of attention obtained using the ANT might benefit from using multiple electrode configurations, targeting multiple nodes implicated in the different attention networks proposed by Posner and Peterson [18].

## Conclusions

Transcranial direct current stimulation directed at the right inferior frontal cortex led to a significant increase in alerting attention in this study. This result is important both to the neuroscience community, as it demonstrates the role of this brain area in the alerting network, as proposed by Posner and Peterson [18], and clinically, as alerting deficits are characteristic of a various neurocognitive disorders. Additionally, this effect was strongly correlated with our previously reported effects of tDCS on object detection. These results suggest that the effects of tDCS on alerting are related to effects of tDCS on hit rate after training, but not on false alarm rate or *d’*. Enhancement of alternative cognitive mechanisms, such as increased neuronal plasticity during training or decreased impulsivity in responding during test, may account for the effects of tDCS on false alarm rate and *d’*.

## Methods

### Participants

Twenty healthy participants gave informed consent and participated in this experiment. One subject performed less than two standard deviations below the mean and was excluded from further analysis. Of the remaining 19 participants (11 male, age = 23.4 yrs, 7.7 yrs SD), 9 were randomly assigned to receive 2.0 mA tDCS, while 10 received 0.1 mA. All participants met the following inclusion criteria: English as a first language, no history of head injuries or concussions resulting in loss of consciousness or hospitalization, right-handedness according to the Edinburgh Handedness Inventory [33], no history of psychiatric or neurological disorders, alcohol or drug abuse, or current medication affecting the CNS, and good or corrected vision and hearing. The study was approved by the Institutional Review Board of the University of New Mexico. The authors declare that they have no conflicts of interest in this research.

### Target object detection task

**Figure 3:**
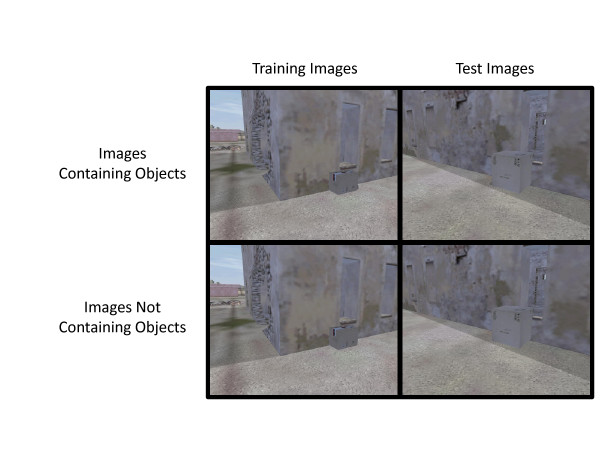
***Object Detection Task Training and Test Stimuli. ***Examples of training (left) and test (right) stimuli are depicted. Images in the top row contain a target object, while those in the lower row do not. In this example, the target object is a small bomb hidden behind the boxes, as indicated by a dark patch.

This experiment was designed to test subject’s ability to learn to detect hidden and camouflaged objects placed in a complex virtual environment. In order to engage and maintain a typical participant’s interest, it was designed to be similar to modern video games, which often include a wartime theme. Five-second video clips from training scenarios from the DARWARS virtual reality training environment were captured for use as feedback in the task [34]. Still images were extracted from these videos and edited to include or remove specific objects. Examples of images presented during training and testing and a full description of the threat detection task can be found in Figure 3, as well as Coffman et al. [11] and Clark et al. [10].

**Figure 4:**
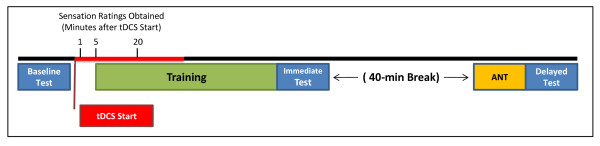
***Timeline of Events. ***Each test period (blue) lasted about 15 min. Training (green) lasted approximately 1 h. Immediate and delayed tests were separated by 1 h. The Attention Networks Test (ANT; orange) was administered immediately before the delay test and lasted approximately 20 min. TDCS (red) was administered starting 5 min before training and lasted for a total of 30 min. Participants were asked to indicate the amount of tDCS-induced sensation that was present at three time points (1, 5 and 20 min after start of tDCS administration).

Participants were first tested for their baseline ability to detect target objects before training, after which participants were trained to detect target objects while receiving either 0.1 mA or 2.0 mA tDCS for 30 minutes. Participants were tested immediately after training was completed (immediate test) and again one hour after the end of training (delayed test; Figure 4). Baseline, immediate, and one-hour delayed tests each consisted of 100 images presented with no feedback, which were different for each phase of testing. Training sessions consisted of four 11-minute blocks of 60 trials, each of which included an image and appropriate audiovisual feedback, with short rest periods between blocks. Each image was presented for two seconds with an inter-trial interval that varied randomly across trials ranging from four to eight seconds. Participants were instructed to scan the images for target objects with no prior information given about the nature of the target objects. Thus, the participant discovered the correct and incorrect responses to each image after examining the audiovisual feedback on each training trial. The one-hour delayed test was designed to examine retention of learned target object detection ability.

### Attention network task

Immediately before the one-hour delayed test, participants completed a 20-minute version of the ANT to assess participant’s alerting, orienting, and conflict resolution measures of attention (Figure 4). The ANT was performed as described by Fan et al. [15], with the exception of response method. Participants responded using the same two fingers and keypad that was used in the target object detection task rather than using the left and right mouse buttons and thumbs. The ANT requires participants to determine the direction (left or right) of a target arrow that appears directly above or below a central fixation that may or may not be accompanied by flankers. These flankers, if present, may point the same direction as the target arrow (congruent) or the opposite direction of the target arrow (incongruent). Additionally, the presentation of the target arrow may or may not be preceded by a temporal or spatial cue, consisting of an asterisk (*). Temporal cues occurred 100 ms before the stimulus and were presented either at the fixation point (center cue) or at both possible target locations (double cue). Spatial cues also occurred 100 ms before the stimulus and were presented at the spatial location of the target arrow.

The alerting effect is calculated by subtracting the mean RT of the double-cue condition from the mean RT of the no-cue condition, as neither of those conditions provide information concerning whether the target would appear above or below the fixation point (spatial information). Without a warning cue (the no-cue condition), attention remains diffused across or oscillating between the upper and lower possible target locations; the double-cue condition impacts attention in the same way, except it alerts the participant to the imminent appearance of the target. The orienting effect is calculated by subtraction of the mean RT of the spatial-cue condition from the mean RT of the center-cue condition. Both of these conditions provide information concerning the impending presentation of a target, but the spatial-cue carries the additional information of target location, allowing subjects to orient attention to the appropriate location prior to target presentation. The executive control effect (conflict resolution) is calculated by subtracting the mean RT of the congruent flanking conditions (all five arrows pointing the same direction) from the mean RT of the incongruent flanking conditions (the target arrow pointing the opposite direction of the flanking arrows).

### Transcranial direct current stimulation

Anodal tDCS was delivered for 30 minutes near 10–10 EEG location F10, over the right sphenoid bone. The location near F10 was suggested from functional magnetic resonance imaging (fMRI) and magnetoencephalography (MEG) studies of changes in brain networks associated with the acquisition of expertise in this task [10]. TDCS was administered through 11 cm^2^ square saline-soaked sponge electrodes. The cathode was placed on the subject’s left upper arm. Electrodes were secured to the scalp and upper arm using Coban self-adherent wrap. TDCS was initiated five minutes before training and continued throughout the first two of four training blocks (Figure 4). We chose this timing because the effects of tDCS have been demonstrated to continue after current administration is ended for at least as long as stimulation was administered [35]. Current was set to either 0.1 mA or 2.0 mA. A current strength of 0.1 mA (the lowest setting on our stimulation device) was selected as our control condition in order to induce physical sensation associated with tDCS (e.g. tingling and/or itching) without stimulating the brain areas beneath. Recent research has shown that traditional methods of “sham“ tDCS stimulation involving ramping the current on and then off after a short (usually 30 seconds) duration may not be as effective in blinding participants to stimulation condition as previously thought [36]. Simulation studies by Miranda et al. [37] suggest that current strengths less than 0.5 mA at the electrode size used in this study have no effect on brain activity in neural tissue 12 mm below the skin surface. In our control condition, participants received less than 20% of this current strength. Additionally, modulation of motor evoked potential amplitude does not seem to occur at current strengths equal to or below 0.2 mA [38], so we are confident that this stimulation condition had little or no effect on the brain.

Both participants and experimenters were blind to the amount of current delivered. Experimenter blinding was accomplished using a coded switch box, with inputs for positive and negative leads from two current generators and outputs for only two electrodes, one anode and one cathode. One current generator was set to 0.1 mA and the other was set to 2.0 mA. A six-way switch interrupted the circuit, with three settings supplying current to the output leads from one current generator, and the remaining three supplying the output from the other current generator. The inputs that were not actively supplying current to the output leads were routed through a simple circuit loop to maintain the activity of the inactive current generator. The six-way switch was coded by a third party to ensure experimenter blinding.

During tDCS, participants were asked to describe their physical sensations at 1, 5, and 20 minutes after the start of tDCS. Subjects were asked to report sensation on three 10-point Likert scales for itching, tingling, and heat. TDCS was stopped if participants reported a seven or higher on any scale, though no subjects reported this level of sensation. As an additional safety measure, and to elucidate possible state-dependent effects of tDCS, mood/state was assessed before and after experimentation using 6-point Likert scales for the following nine measures: (1) I feel nervous or excited. (2) I feel tired or fatigued. (3) I feel confused or disoriented. (4) I feel sad or down. (5) I feel tense or frustrated. (6) I feel dizzy or light-headed. (7) I feel nauseous. (8) Physically, I feel pain or discomfort. (9) I feel unable to concentrate or pay attention.

### Data analysis

We first compared participants’ ANT scores for alerting, orienting, and executive attention using a 2 (tDCS condition: 0.1 mA vs 2.0 mA) x 3 (alerting vs orienting vs executive attention) ANOVA. Post-hoc comparisons between tDCS conditions were made using individual Student’s t-tests, with a Bonferroni corrected α = 0.017 (0.05/3). We then used Pearson correlation to assess the relationship between attention measures identified as significantly affected by tDCS (alerting was the only such measure) and measures of signal detection (*d’,* β *,* hits and false alarms) in the target object detection task. Correlation coefficients were computed separately for participants in receiving 0.1 mA or 2.0 mA tDCS. *d’* and β were calculated based on the hits (correct responses to images containing target objects) and false alarm (incorrect responses to images not containing target objects) rates according to calculations described by Stanislaw and Todorov [39]. Briefly, signal detection (d’) is calculated by subtracting the z-normalized false alarm rate from the z-normalized hit rate, while response bias (β) is calculated by raising e to the power of ½ the difference between the squared, z-normalized false alarm rate and the squared, z-normalized hit rate. Student’s *t*-test was used to compare tDCS-induced sensation and mood/state between groups and Pearson correlation was used to examine the relationship between sensation, measures of mood/state, and measures of attention identified as significantly affected by tDCS.

## Competing interests

The authors declare that they have no competing interests.

## Authors’ contributions

BC conceived the study, participated in design of the study, assisted with data collection, performed data analysis, and drafted the manuscript. MT participated in design of the study, assisted with data collection, and helped to draft the manuscript. VC participated in design of the study, helped to coordinate the study, managed experimenter blinding procedures, and helped to draft the manuscript. All authors read and approved the final manuscript.
